# A retrospective cohort study of a PIRFAS- and ultrasound-guided program for pressure ulcers in prone orthopedic surgery

**DOI:** 10.3389/fmed.2025.1668492

**Published:** 2026-04-10

**Authors:** Lijie Yuan, Ying Fu

**Affiliations:** Department of Orthopedic, The Affiliated Cancer Hospital of Harbin Medical University, Harbin, Heilongjiang, China

**Keywords:** orthopedic procedures, perioperative nursing, pressure ulcer, prone position, ultrasonography

## Abstract

**Objective:**

Pressure ulcers are recognized as a prevalent intraoperative complication in prone-positioned orthopedic surgery patients. This study aimed to explore the effects of a gradient warning nursing procedure (GWNP) guided by the Pressure Injury Risk Factor Assessment Scale (PIRFAS) and ultrasonography on pressure ulcers in prone-positioned orthopedic operation patients.

**Methods:**

This retrospective cohort study enrolled 126 patients who underwent prone-positioned orthopedic surgery at our hospital between May 2022 and May 2024. The assignment to groups was based on a hospital-wide implementation of a new nursing protocol on 1 June 2023. Accordingly, 60 patients admitted before this date constituted the regular group and received regular care, while 66 patients admitted on or after this date constituted the joint group and received the gradient early warning nursing procedure guided by the Pressure Injury Risk Factor Assessment Scale (PIRFAS) and ultrasonography, in addition to regular care. The incidence of pressure ulcers, pressure ulcer grading, number and area of injury, simplified comfort status scale (GCQ) pre- and post-care, hospitalization duration, and costs were collected and compared between the two groups. Statistical analyses were performed using SPSS 25.0, with the *χ*^2^ test, independent samples *t*-test, Mann–Whitney U-test, and multivariate regression analysis being employed as appropriate for data types and comparison purposes.

**Results:**

The results in our study revealed that the incidence (adjusted odds ratio [aOR: 0.11], 95% CI: 0.02–0.48), number (adjusted mean difference [aMD: −0.82], 95% confidence interval [CI]: −1.04 – −0.60), area (aMD: −0.62, 95% CI: −0.83 – −0.41), and grading of pressure ulcers were significantly decreased in the joint group versus the regular group (*p* < 0.05), indicating the implementation of the gradient early warning nursing procedure guided by the PIRFAS and ultrasonography effectively reduced the incidence and tissue damage severity. There was no statistically significant difference in the comparison of GCQ scores of dimensions prior to nursing in the two groups (*p*>0.05), whereas the GCQ scores regarding physiological (aMD: 3.32, 95% CI: 2.45–4.19) and psycho-spiritual (aMD: 2.23, 95% CI: 1.38–3.08) aspects in the joint group post nursing were evidently higher versus the regular group (*p* < 0.05), with the difference insignificant in environmental and socio-cultural scores. The hospitalization duration (aMD: −4.50 days, 95% CI: −6.12 – −2.88) and costs (aMD: −2.00 ten thousand yuan, 95% CI: −2.45 – −1.55) of the joint group were prominently decreased versus the regular group, with statistical significance (*p* < 0.05).

**Conclusion:**

The GWNP guided by the PIRFAS and ultrasonography could effectively represent an effective bundled intervention for prone-positioned orthopedic surgery. Implementation of this protocol in clinical practice can potentially enhance patient safety by reducing pressure ulcer incidence and severity while also improving healthcare resource utilization. Future research should focus on validating these findings through multicenter randomized controlled trials.

## 1 Introduction

Pressure ulcers are local damage to the skin and/or subcutaneous tissues resulting from pressure or pressure combined with shear forces, inadequate tissue nutrient supply, impaired blood circulation, loss of normal skin function, and tissue breakdown or necrosis (1, 2). Evidence indicates that pressure ulcers are not uncommon among hospitalized patients, with surgical patients facing a particularly elevated risk (3). During surgery, due to the fixed position, especially in the prone position, the probability of acute intraoperative pressure ulcers is markedly increased due to the long operation duration, surgical stress, and other factors (4, 5). The presence of pressure ulcers will aggravate the pain of patients and treatment burden, prolong the hospitalization duration, increase the risk of nursing care, dissatisfy patients and families, and lead to medical disputes (6).

Perioperative nursing is a critical part of clinical nursing, with the incidence of intraoperative pressure ulcers being an important indicator for the quality of care (7). The gradient warning nursing procedure (GWNP) represents a novel clinical care model that effectively reduces the incidence of hospital-acquired pressure injuries by stratifying patients into distinct risk levels and implementing targeted preventive interventions (8). Studies have documented that integrating comprehensive perioperative nursing care with the implementation of a standardized pressure injury risk assessment scale significantly reduces pressure ulcer incidence (9). In decades, due to its reproducibility, dynamics, real-time nature, and rapidity, ultrasonography has been applied to the assessment and treatment of pressure ulcers in the clinic (10). Specific sonographic criteria, such as inhomogeneous hypoechoic areas and discontinuous fascial lines, have been validated for identifying early deep tissue injury (11, 12). Furthermore, semi-quantitative Doppler grading (e.g., the Adler scale) provides a reliable assessment of local tissue perfusion, aiding in risk stratification (13). This imaging-based approach permits the early detection and staging of tissue damage, which guides preventive and therapeutic nursing measures, assesses treatment efficacy, and helps formulate comprehensive pressure ulcer management strategies (14). The application of ultrasound for pressure ulcer preventive care was reported to significantly reduce the progression of pressure ulcers (15). However, there is a paucity of the application of a GWNP co-directed by the PIRFAS and ultrasound to pressure ulcer formation in patients with orthopedic surgery in the prone position. The rationale for combining these modalities lies in their complementary roles: while PIRFAS provides a comprehensive assessment of systemic and clinical risk factors, ultrasonography offers an objective, real-time evaluation of localized tissue status and microcirculation. This combined approach aims to bridge the gap between identifying at-risk patients and detecting subclinical tissue damage before it progresses to visible ulcers. Although this specific combined protocol is novel, its face validity is supported by preliminary evidence showing that risk assessment scales can effectively stratify patients, and ultrasound can accurately detect early tissue changes indicative of pressure ulcers (16, 17).

In this study, this retrospective cohort study aimed to evaluate the effects of a GWNP guided by the PIRFAS and ultrasound on pressure ulcer formation in prone-positioned orthopedic operation patients in terms of the incidence, pressure ulcer grading, number and area of injury, and simplified GCQ prior to and post care, hospitalization duration, and hospitalization costs, which might provide an evidence-based basis for clinical practice.

## 2 Materials and methods

### 2.1 Statement of ethics

The present study was approved by the Ethics Committee of the Affiliated Cancer Hospital of Harbin Medical University (approval No.: 2024-HHL-0032; date of approval: 18 February 2024). Given that this study was retrospective and only de-identified patient data were used, informed consent was not required since there were no risks or adverse effects on patient care. This waiver is in line with regulatory and ethical guidelines related to retrospective studies.

### 2.2 Study design

This retrospective cohort study enrolled 150 patients undergoing prone-position orthopedic surgery at our hospital between May 2022 and May 2024. A hospital-wide nursing protocol mandating the use of the PIRFAS- and ultrasound-guided GWNP was implemented on 1 June 2023. Consequently, patients admitted before this date (*n* = 60) constituted the regular group (receiving regular care), and those admitted on or after this date (*n* = 66) constituted the joint group (receiving the GWNP in addition to regular care). The sampling period (May 2022 to May 2024) was chosen to provide an adequate sample size and to capture patient data both before and after the implementation of the new nursing protocol on 1 June 2023. This timeline allowed for a natural comparison between patients who received regular care during the earlier phase and those who received the new PIRFAS- and ultrasound-guided intervention during the later phase.

### 2.3 Inclusion and exclusion criteria

The inclusion criteria were as follows: (1) patients whose prone orthopedic surgery was operated on in our hospital; (2) patients who had no skin injury before surgery, skin integrity, and no pressure ulcer condition; (3) patients who were aged ≥18 years old, regardless of gender; and (4) patients who provided complete clinical data.

The exclusion criteria were as follows: (1) preoperative presence of dermatological changes, such as skin inflammation and neurodermatitis. Rationale: to ensure baseline skin integrity; (2) skin injuries caused by direct and sustained pressure from medical devices (e.g., monitor leads, electrocautery cords, surgical frames, or fixation devices) during surgery, which were identified upon immediate postoperative examination and showed a shape conforming to the device. The exclusion of any such case was adjudicated by an attending physician not involved in the patient’s care and a specialized wound care nurse to reach a consensus. Rationale: to isolate posture-related pressure injuries; (3) emergency surgery. Rationale: to control for physiological instability as a confounding factor; (4) advanced stage of tumors or severe dysfunction of major organs (cardiac, pulmonary, and renal). Rationale: to minimize the impact of impaired tissue perfusion and healing; (5) patients with combined psychiatric disorders. Rationale: to ensure reliable self-reporting of comfort scores; and (6) the presence of uncontrollable hypertension and other diseases unable to be treated with surgery. Rationale: to control for high-risk systemic confounders.

A total of 150 patients meeting the basic criteria (prone-position orthopedic surgery, age ≥18 years, and no pre-existing skin injury) were initially identified through the electronic medical record system between May 2022 and May 2024. Overall, 24 patients were excluded for the following reasons: incomplete clinical data (*n* = 12), preoperative dermatopathological changes (*n* = 5), intraoperative device-related pressure injuries (*n* = 2), emergency surgery (*n* = 3), comorbid psychiatric disorders (*n* = 1), and severe multiple organ dysfunction (*n* = 1). Cases with any missing data were excluded from the final analysis. Consequently, the final analysis included 126 patients with complete datasets (60 in the regular group and 66 in the joint group).

### 2.4 Methods of care

All surgical procedures for both patient groups were performed by a single, consistent team of orthopedic surgeons and anesthesiologists. This standardization ensured that variations in surgical technique, anesthesia management, and overall perioperative care were minimized, thereby eliminating the potential for clustering effects by individual care providers.

The regular group was given routine care, mainly including: (1) routine implementation of the basic principles and requirements of surgical position placement; (2) use of sponge pads, cloth pads, or of the suspension method for the compressed parts of the bony prominence; (3) strict implementation of intraoperative patients’ heat preservation measures, paying attention to the non-surgical part of the body cover during the operation, the rinsing fluid warmed up to 36 °C – 37 °C, and the conditions of giving warmed blood transfusion or infusion, and strictly preventing the patients from hypothermia during the operation; and (4) keeping the surgical towel dry and flat to prevent skin disinfectant and rinsing solution from flowing to the pressure area.

The joint group adopted the gradient early warning nursing program guided by the PIRFAS and ultrasonography on the basis of the regular group; the methods are as follows: (1) based on the PIRFAS- and ultrasound-guided assessments: ① based on the PIRFAS assessment, The Waterlow Pressure Ulcer Risk Factor Evaluation Scale was used to assess the risk level of pressure ulcers. The Waterlow Assessment Scale consists of nine major items: gender and age, body shape, weight and height, skin type, bowel control, exercise capacity, appetite, cardiovascular and systemic conditions, nutritional deficiencies, and medication. Each item is subdivided into three to seven levels with corresponding scores. Preoperative assessment was performed according to the condition of the patient, and then the scores were aggregated, with a score of < 10 as no risk, 10–14 as grade I (low risk), 15–19 as grade II (moderate risk), and 20 and above as grade III (high risk), indicating that the higher the scores, the higher the likelihood of pressure ulcers occurring (18). ② Ultrasound assessment: All ultrasonographic examinations were performed by two senior sonographers with specialized training in musculoskeletal imaging, using a high-frequency linear array transducer (Philips EPIQ 7G) with a preset musculoskeletal scanning mode (frequency: 12 MHz). A standardized scanning protocol was applied to predefined bony prominence sites (bilateral occipital protuberances, scapulae, sacrum, and heels). Each site was scanned in both longitudinal and transverse planes using B-mode to assess tissue structure and color Doppler to evaluate blood flow according to the Adler grading system. During the examination, the sites prone to pressure ulcers were adequately exposed and routinely ultrasonographically examined. Ultrasound evaluation: the sites prone to ulcer incidence were fully exposed, and routine ultrasound scanning was performed to obtain relevant ultrasound information: blood flow distribution, elasticity score, boundary and echo, etc. If the ultrasound results showed the presence of the following ultrasound manifestations, it indicated the presence of deep tissue damage: inhomogeneous hypoechoic areas, discontinuous fascial lines, hypoechoic foci, and unclear subcutaneous tissue hierarchies.

The Adler blood flow grading criteria were also referenced to semi-quantitatively assess local tissue perfusion at potential pressure sites. The grading was defined as follows: Grade III: more than four visible vessels with abundant flow signals; Grade II: several small vessels or one major vessel visible; Grade I: one or two punctate flow signals with scant flow; and Grade 0: no detectable blood flow (19). To ensure consistency, both sonographers independently analyzed the images for the first 20 enrolled patients, demonstrating excellent inter-rater reliability (Cohen’s kappa = 0.85).

(2) Nursing records: ① based on the usual protective measures, polymer polyurethane gel position pads for the local skin at the bone protrusion and local pressure paste Amp paste gel dressings were used to increase the compression resistance of the support point and reduce the vibration and dispersion of the pressure; ② for the patients with moderate risk assessed by the PIRFAS and/or ultrasound assessment, a stable prone position was maintained with limbs stretched, a soft cushion was used for supporting the articulation part of the depression; a square one-piece anti-pressure cushion was applied; the position of the cushion and the skin between the wrinkles was maintained to avoid skin extrusion; the restraining belts were kept soft and smooth, appropriately elastic, and lined with soft cloth pads to avoid rough surfaces, if necessary; to reduce the number of times and the angle of bed shaking during the operation, the tilt angle was appropriately controlled between 10° to 20° to avoid the body shifted; If conditions permitted, the bony prominence was gently massaged or the pressurized area was gently lifted to relieve local pressure every 1 h–2 h; and ③ for the patients with a severe risk tested by the PIRFAS and/or ultrasound assessment, the related results need to be reported to the quality control team of the nursing department. The quality control team personnel reviewed the accuracy of the nurse’s assessment content according to the actual situation of the patient, checked the relevance, effectiveness, and comprehensiveness of the protective measures to be taken, and finally made a conclusion or provided guidance and feedback to the department. Thereafter, the department adjusted the nursing interventions with the feedback and finally eliminated the occurrence of intraoperative pressure ulcers. To ensure protocol adherence, an intervention checklist was used. Compliance was audited by an independent operating room head nurse. The adherence rates were as follows: use of gel pads and dressings: 100%; positional adjustments for moderate-risk patients every 1–2 h: 98.5%; and reporting to the quality control team for all high-risk patients: 100%. This demonstrates high fidelity to the intervention bundle. Patients in both groups were cared for until discharge. The difference in the nursing care in the two groups is displayed in Table 1.

**Table 1:** Comparison of nursing care between the regular and joint groups.

Intervention component	Regular group	Joint group
Pressure relief	Use of sponge/cloth pads or suspension method for bony prominences	- Polymer polyurethane gel position pads; Amp paste gel dressings at pressure points
Risk assessment	None	Waterlow scale Ultrasound evaluation
Positioning	Standard positioning	- Customized positioning based on risk level - Square anti-pressure cushions - Controlled bed tilt (10–20°) - Position checks every 1–2 h
Special interventions	None	Moderate risk: hourly pressure relief; high risk: nursing quality control team review; and protocol adjustments based on feedback
Monitoring	Routine observation	Continuous ultrasound monitoring of high-risk areas

### 2.5 General characteristics data collection

The general demographic data of patients in the two groups were retrospectively collected through the electronic medical record system, mainly including gender, age, body mass index (BMI), presence of comorbidities, type of surgery, duration of surgery, amount of intraoperative bleeding, and the use of perioperative medications known to potentially influence tissue perfusion and bleeding risk, specifically anticoagulant and antiplatelet agents.

### 2.6 Indicators related to pressure ulcers

The occurrence of pressure ulcers in the two groups was retrospectively collected through the electronic medical record system, with the documentation of lesion location, number, and area. The observation period for pressure ulcer occurrence spanned from the initiation of surgery until patient discharge. No post-discharge surveillance was conducted. Referring to the grading standards of the National Pressure Ulcer Advisory Panel (NPUAP), the specific grading standards are as follows: intact skin with the presence of non-blanchable erythema and white erythema in a very small part of the skin indicative of grade 1; partial-thickness skin loss with part of the exposed dermis indicative of grade 2; full-thickness skin loss, with exposed adipose and granulation tissues, and localized carrion and burnt scabs indicative of grade 3; and full-thickness skin loss, with exposed cartilage, ligament, and bone indicative of grade 4 (20). The incidence rate of pressure ulcers is calculated as follows: number of patients with pressure ulcers/total number of enrolled patients × 100%. Although the joint group received continuous ultrasound monitoring intraoperatively as part of the intervention, the primary outcome of pressure ulcer diagnosis was assessed postoperatively using the same clinical method for both groups. These final assessments were conducted by ward nurses who were blinded to the patient’s group assignment, thereby minimizing potential detection bias. It should be noted that ultrasonography for pressure ulcer assessment is not part of routine postoperative care in Chinese hospitals; it was performed specifically for this study to obtain objective measures of tissue damage.

### 2.7 Comfort

The GCQ scoring data were retrospectively collected through the electronic medical record system in two groups prior to and post care. The GCQ is a 28-item instrument divided into four subscales: physiological (5 items; subscale range: 5–20), psycho-spiritual (11 items; subscale range: 11–44), environmental (6 items; subscale range: 6–24), and socio-cultural (6 items; subscale range: 6–24) subscales. Each item is scored on a 4-point Likert scale, resulting in a total score ranging from 28 to 112, with higher scores indicating greater comfort. The GCQ has demonstrated good reliability and validity in surgical populations, with reported Cronbach’s *α* values between 0.87 and 0.89 (21). The GCQ was administered specifically for this study. In standard Chinese hospital practice, patient comfort questionnaires are not routinely administered or documented after each surgical procedure.

### 2.8 Hospitalization indicators

The duration and costs of hospitalization in two groups were retrospectively collected through the electronic medical record system.

### 2.9 Sample size estimation and power analysis

The sample size for this retrospective cohort study was determined based on the availability of complete electronic medical records for patients meeting the inclusion criteria between May 2022 and May 2024. Given the exploratory nature of this study and the lack of prior effect size estimates for the combined intervention, a formal *a priori* sample size calculation was not conducted. However, a post-hoc power analysis was performed using G*Power 3.1. With a significance level of *α* = 0.05 (two-tailed) and an effect size d of 0.6, the achieved power (1-*β*) for the total sample of 126 patients (60 in the regular group and 66 in the joint group) was 0.92, indicating that the current sample size provided sufficient statistical power to support the inference regarding the primary outcomes.

### 2.10 Statistical methods

SPSS 25.0 statistical software was used to analyze the data, and the chi-squared test was used to express counting information as *n* or %. The measurement information was primarily tested by the Shapiro–Wilk method to assess the conformity of the normal distribution. Data conforming to normal distribution were expressed as mean ± standard deviation (SD), and intergroup comparisons were performed using the independent samples *t*-test. Data not conforming to normal distribution were expressed as median (P25, P75), and the Mann–Whitney U-test was employed for comparisons between the two groups.

To estimate the independent effect of the nursing intervention while controlling for potential confounding, we conducted a series of prespecified multivariable regression analyses for all primary and secondary outcomes. The key confounding variables, identified *a priori* based on clinical knowledge, included age, body mass index (BMI), the presence of any comorbidities, type of surgery, surgical duration, intraoperative bleeding volume, and the use of anticoagulant or antiplatelet medication. The specific regression models were used according to the nature of the outcome variable: a multivariate logistic regression analysis was used for the binary outcome (incidence of pressure ulcer), an ordered multivariate logistic regression analysis was used for the ordered classification outcome (grading of pressure ulcer), and a multivariate linear regression analysis was used for the indicators conforming to the normal distribution in the continuous outcome (number/area of ulcers and scores of each dimension of GCQ). The length of hospital stay and hospitalization expenses with non-normal distribution were analyzed using a multivariate linear regression analysis after logarithmic transformation combined with quantile regression. All analyses fully reported the adjusted effect estimate (adjusted OR/MD) and 95% confidence interval (CI). For the regression models, the Hosmer–Lemeshow goodness-of-fit test was used, indicating satisfactory model calibration (*p* > 0.05). Differences were considered statistically significant at a *p*-value of < 0.05.

## 3 Results

### 3.1 Comparison of general characteristics of patients in two groups

Comparison of gender, age, BMI, comorbidity, type of surgery, operation duration, intraoperative bleeding, and use of anticoagulant/antiplatelet medication in the two groups did not differ statistically significant (*p* > 0.05), suggesting that the groups are comparable (Table 2).

**Table 2:** Comparison of general characteristics of patients in the two groups.

Index		Regular group (*n* = 60)	Joint group (*n* = 66)	Absolute difference (95% CI)	*χ*^2^/*t*/*Z* value	*p-*value
Gender (n)	Male	32	37	1.12 (0.55–2.25)	0.094	0.759
	Female	28	29			
Age (M ± SD, years)		62.24 ± 4.86	61.80 ± 5.25	0.48 (−1.31–2.26)	0.526	0.600
BMI (M ± SD, kg/m^2^)		21.01 ± 3.50	22.08 ± 3.47	−1.07 (−2.30–0.16)	1.722	0.088
Any comorbidities (*n*)	Yes	10	14	1.35 (0.55–3.31)	1.226	0.268
	No	50	42			
Type of surgery (*n*)	Cervical spine surgery	24	28	0.07 (0.02–0.24)	0.264	0.876
	Lumbar spine surgery	20	23			
	Thoracic spine surgery	16	15			
Surgical duration (M ± SD, h)		3.12 ± 0.68	3.30 ± 0.75	−0.23 (−0.48–0.02)	1.406	0.162
Intraoperative bleeding (M ± SD, mL)		90.38 ± 7.74	90.85 ± 7.88	−0.47 (−3.23–2.29)	0.337	0.737
Anticoagulant/antiplatelet Use (*n*)	Yes	9	11	0.16 (0.42–3.23)	0.065	0.798
	No	51	55			

BMI, body mass index. Data are presented as n (%) for categorical variables or mean ± standard deviation (M ± SD) for continuous variables. Categorical variables were compared using the chi-squared (*χ*^2^) test, and continuous variables conforming to normal distribution were compared using the independent samples *t*-test. The absolute difference (95% CI) for categorical variables is presented as odds ratio (95% CI), and the absolute difference for continuous variables is presented as mean difference (95% CI). A *p*-value of < 0.05 was considered statistically significant.

### 3.2 Comparisons of the incidence of pressure ulcers between the two groups

The incidence rate of pressure ulcers is an intuitive index for evaluating the pressure-induced ulceration. The incidence rate of pressure ulcers of the joint group (3.03% vs. 23.33%) was evidently decreased compared to the regular group, with the difference statistically significant (*p* < 0.05), thus indicating that the adoption of the PIRFAS alongside the ultrasound-guided Gradient Early Warning Nursing Program effectively reduces the incidence of pressure ulcers, as shown in Table 3.

**Table 3:** Comparison of the incidence of pressure ulcers in the two groups of patients.

Pressure ulcer site	Regular group (*n* = 60)	Joint group (*n* = 66)	Absolute difference (95% CI)	*χ*^2^ value	*p-*value	Adjusted OR (95% CI)	Adjusted *p*
Head and face (*n*)	4	1					
Iliac crest (*n*)	3	1					
Anterior chest (*n*)	3	0					
Hoof (*n*)	2	0					
Toe (*n*)	2	0					
Ulceration rate (%)	14 (23.33)	2 (3.03)	0.05 (0.22–0.40)	11.686	0.001	0.11 (0.02–0.48)	0.003

Data are presented as *n* (%). The incidence of pressure ulcers between the groups was compared using the chi-squared (*χ*^2^) test. The adjusted odds ratio (aOR, 95% CI) was calculated using a multivariate logistic regression analysis, adjusting for age, BMI, comorbidity burden, surgical type, surgical duration, intraoperative blood loss, and the use of anticoagulant or antiplatelet medication. A *p*-value of < 0.05 was considered statistically significant.

### 3.3 Comparison of pressure ulcer grading between the two groups

Pressure ulcer grading was used to assess the severity of pressure ulcers in patients, with a higher pressure ulcer grading indicating the higher severity of pressure ulcers. The pressure ulcer grading of the joint group (64:1:1:0:0 vs. 46:5:4:3:2) was lower than that of the regular group, with the difference statistically significant (*p* < 0.05), which suggested that the adoption of the PIRFAS and the ultrasound-guided Gradient Early Warning Nursing Program could effectively reduce the severity of pressure ulcers (Table 4).

**Table 4:** Comparison of pressure ulcer grading between the two groups (n).

Pressure ulcer classification	Regular group (*n* = 60)	Joint group (*n* = 66)	Absolute difference (95% CI)	*χ*^2^ value	*p-*value	Adjusted OR (95% CI)	Adjusted *p*
No pressure ulcers	46 (76.67)	64 (96.97)	0.05 (0.19–0.41)	9.891	0.042	0.20 (0.04–0.98)	0.047
Level 1	5	1					
Level 2	4	1					
Level 3	3	0					
Level 4	2	0					

Data are presented as *n* (%). The incidence of pressure ulcers between the groups was compared using the chi-squared (*χ*^2^) test. The adjusted odds ratio (aOR, 95% CI) was calculated using a multivariate logistic regression analysis, adjusting for age, BMI, comorbidity burden, surgical type, surgical duration, intraoperative blood loss, and the use of anticoagulant or antiplatelet medication. A *p*-value of < 0.05 was considered statistically significant.

### 3.4 Comparisons of the number and the area of injuries between the two groups

**Figure 1:**
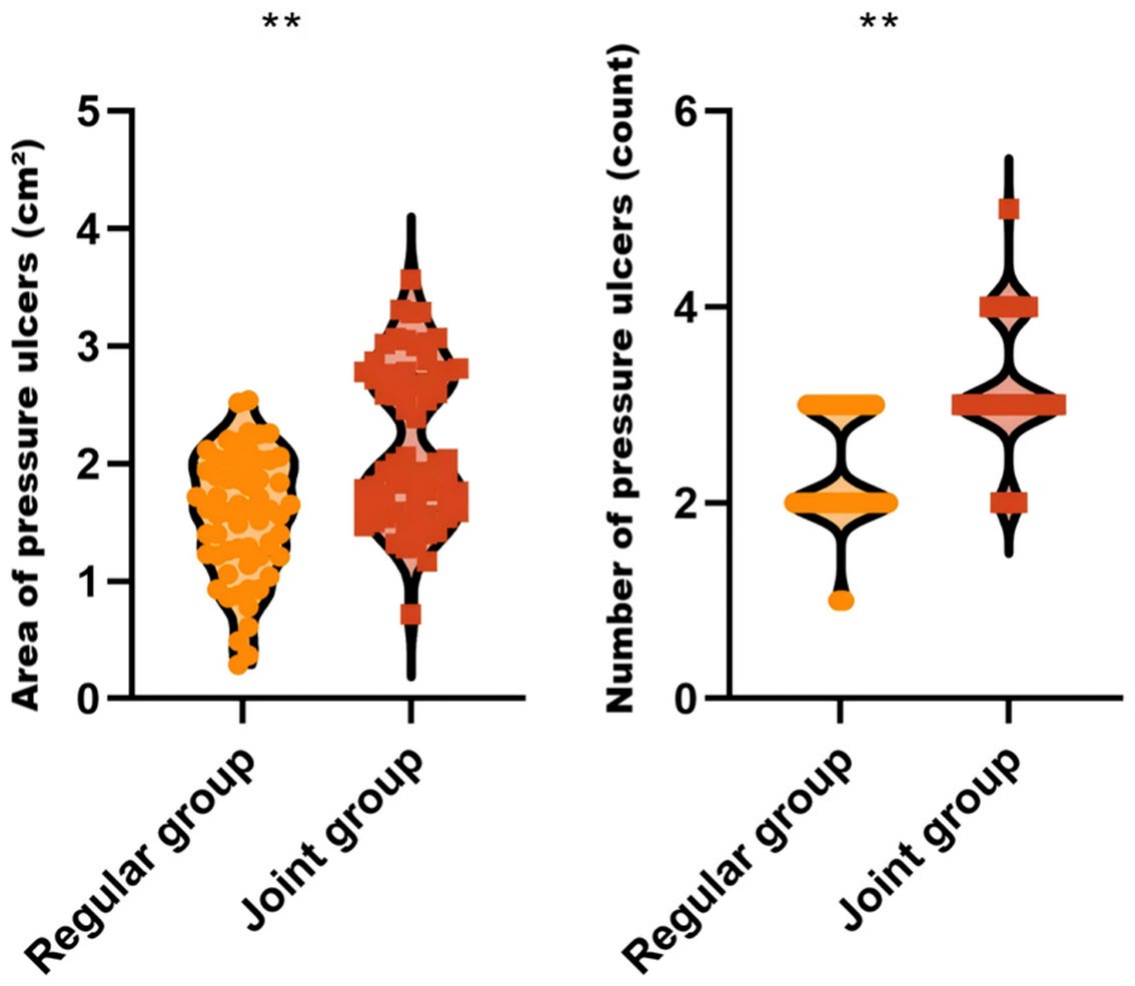
Comparisons of the areas and the number of injuries between the two groups. ** *P* < 0.01.

The severity of pressure ulcers was evaluated by measurement of the number and the areas of ulcers, where a higher count and larger total area indicate greater severity. As shown in Figure 1, compared to the regular group, the joint group showed a significant reduction in both the areas of ulcers (1.56 ± 0.50 vs. 2.18 ± 0.67; adjusted MD = −0.62, 95% CI: −0.83– −0.41) and the number of ulcers (2.35 ± 0.58 vs. 3.17 ± 0.65; adjusted MD = −0.82, 95% CI: −1.04– −0.60) (*p* < 0.05), indicating that the PIRFAS- and ultrasound-guided GWNP effectively mitigate the severity of pressure ulcers in patients undergoing orthopedic surgery in the prone position.

### 3.5 Comparison of GCQ scores prior to and postoperation nursing care between the two groups of patients

The GCQ score was used to test patient comfort, with higher GCQ scores indicative of greater comfort levels. The comparison of GCQ scores between the two groups prior to the nursing care exhibited no statistically significant differences in any of the measured parameters (*p* > 0.05), including physiological (8.20 ± 1.78 vs. 8.35 ± 1.84), psycho-spiritual (18.35 ± 2.26 vs. 18.40 ± 2.37), environmental (13.42 ± 2.92 vs. 13.58 ± 3.34), and socio-cultural (13.76 ± 2.25 vs. 13.98 ± 2.34) parameters, as shown in Table 5.

**Table 5:** Comparison of pre-care GCQ scores between the two groups (M ± SD).

Index	Time	Regular group (*n* = 60)	Joint group (*n* = 66)	Absolute difference (95% CI)	*t-*value	*p-*value	Adjusted MD (95% CI)	Adjusted *p*
Physiological	Pre-operation care	8.20 ± 1.78	8.35 ± 1.84	0.15 (−0.52–0.82)	0.464	0.643	0.14 (−0.53–0.81)	0.672
Psycho-spiritual	Pre-operation care	18.35 ± 2.26	18.40 ± 2.37	0.05 (−0.78–0.88)	0.121	0.904	0.04 (−0.79–0.87)	0.920
Environmental	Pre-operation care	13.42 ± 2.92	13.58 ± 3.34	0.16 (−0.92–1.24)	0.285	0.776	0.15 (−0.93–1.23)	0.781
Socio-cultural	Pre-operation care	13.76 ± 2.25	13.98 ± 2.34	0.22 (−0.56–1.00)	0.537	0.592	0.21 (−0.57–0.99)	0.594

GCQ = simplified comfort status scale. Data are presented as mean ± standard deviation (M ± SD). Intergroup comparisons of pre-care GCQ scores were performed using the independent samples *t*-test. The adjusted mean difference (aMD, 95% CI) was calculated using a multivariate linear regression analysis, adjusting for age, BMI, comorbidity burden, surgical type, surgical duration, intraoperative blood loss, and the use of anticoagulant or antiplatelet medication. A *p*-value of < 0.05 was considered statistically significant.

**Figure 2:**
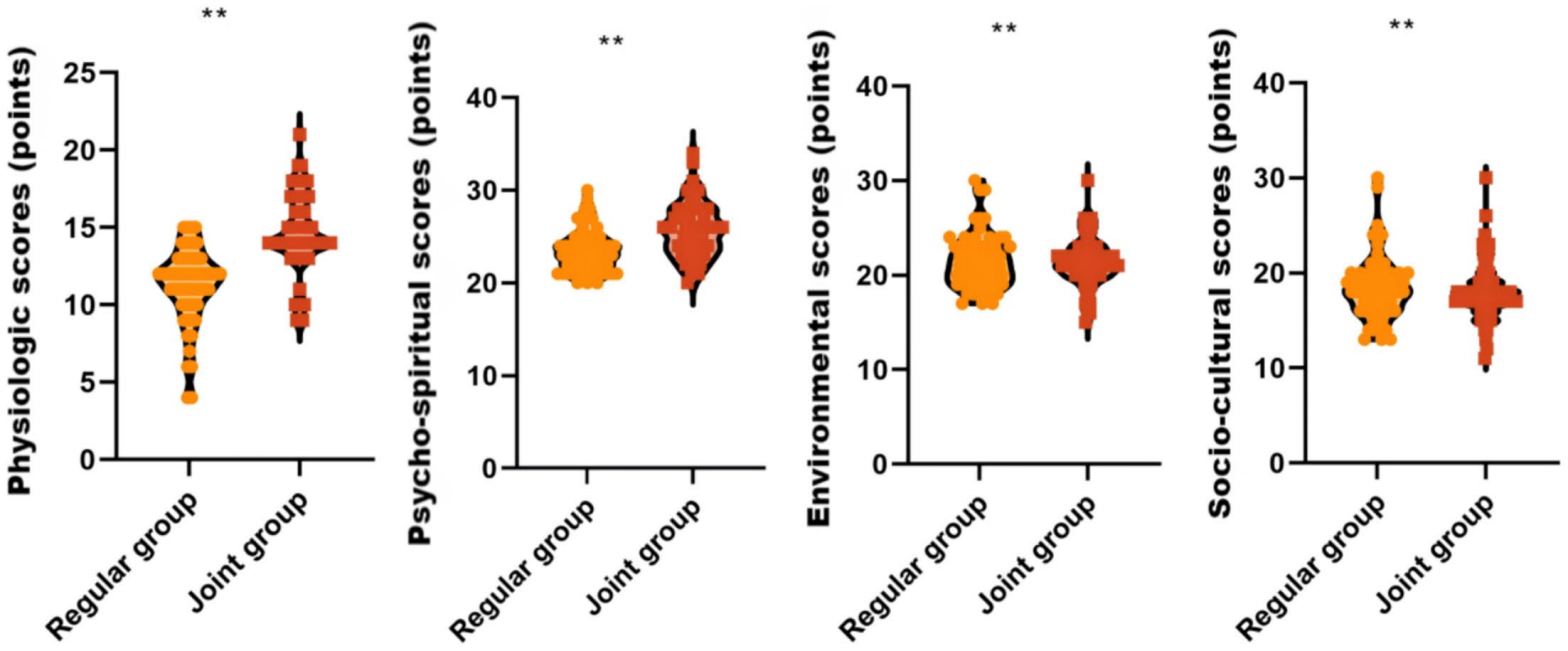
Comparison of GCQ scores between the two groups postoperation nursing care. GCQ is a simplified comfort status scale. ** *P* < 0.01.

The GCQ scores of physiological (14.58 ± 2.53 vs. 11.26 ± 2.40; adjusted MD = 3.32, 95% CI: 2.45–4.19) and psycho-spiritual (25.68 ± 2.92 vs. 23.45 ± 2.26; adjusted MD = 2.23, 95% CI: 1.38–3.08) aspects were significantly higher in the joint group than the regular group postoperation nursing care (*p* < 0.05), whereas the GCQ scores of the environmental (21.06 ± 2.37 vs. 20.98 ± 2.45; adjusted MD = 0.07, 95% CI: −0.76–0.90) and socio-cultural (17.44 ± 2.20vs.17.65 ± 2.35; adjusted MD = −0.22, 95% CI: −1.06–0.62) aspects were not significantly different (*p* > 0.05), thus indicating that the adoption of PIRFAS and ultrasound-guided GWNP could enhance the comfort of patients with prone orthopedic surgery, as shown in Figure 2.

### 3.6 Comparison of hospitalization duration and costs between the two groups

**Figure 3:**
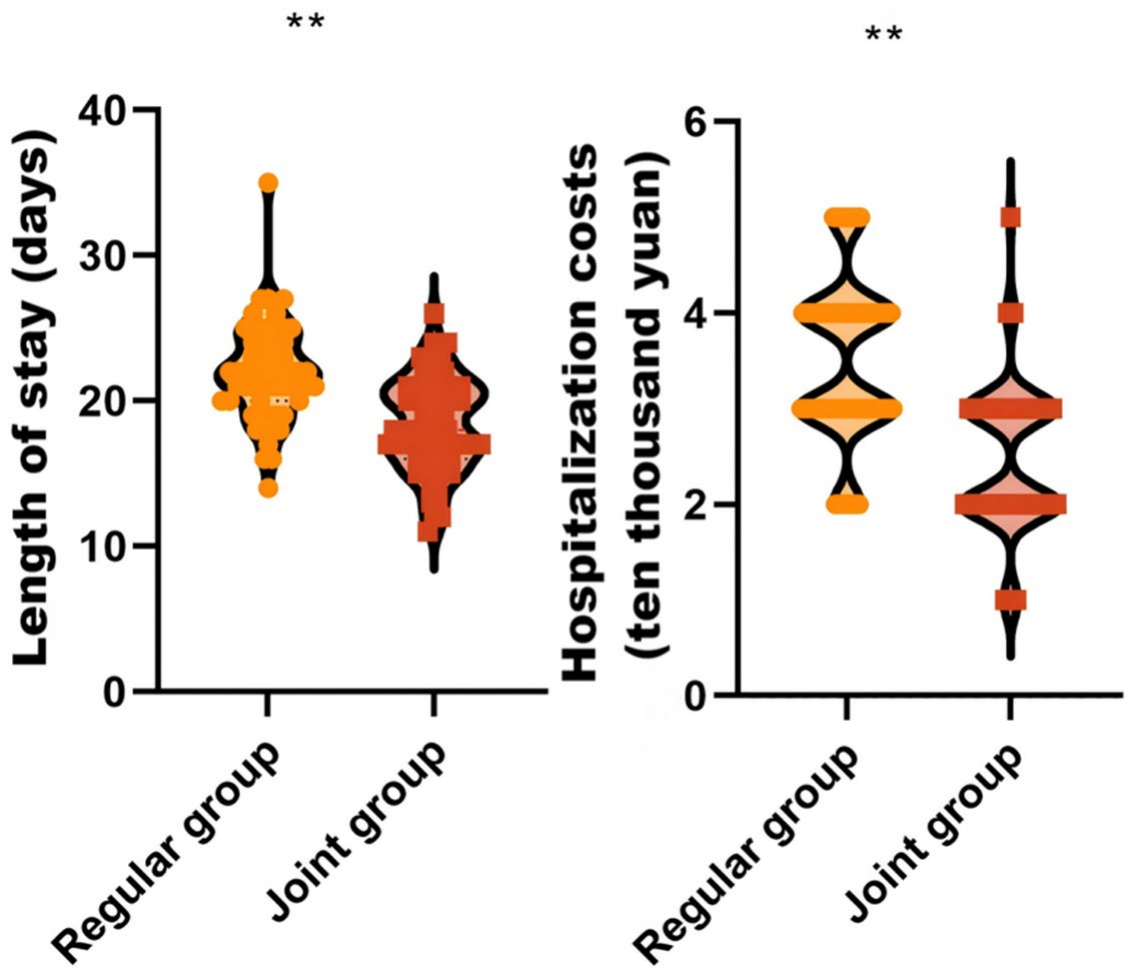
Comparison of hospitalization duration and costs between the two groups. ** *P* < 0.01.

Hospitalization duration serves as a prognostic indicator, where prolonged stays correlate with poorer prognosis. Similarly, total hospitalization costs reflect the financial burden, with high expenses indicating greater medical care-related costs. The joint group demonstrated significantly shorter hospitalization duration [18.00 (16.00, 21.00) vs. 22.50 (20.00, 24.00) days; adjusted MD = -4.50, 95% CI: −6.12–-2.88] and lower hospitalization costs [2.00 (2.00, 3.00) vs. 4.00 (3.00, 4.00) ten thousand yuan; adjusted MD = -2.00, 95% CI: −2.45–-1.55] versus the regular group, which implied that the implementation of the PIRFAS combined with the ultrasound-guided graded early warning care protocol significantly reduces the length of hospitalization and therapy costs in patients undergoing orthopedic surgery in the prone position, as shown in Figure 3.

## 4 Discussion

Pressure ulcers are a prevalent clinical complication, occurring in the coccyx, scapular eminence, femoral trochanter, and other locations of the body, which not only affects the treatment and rehabilitation of the original disease but also further increases physical and mental pain (2). The majority of orthopedic surgeries, especially spinal operations frequently performed in the prone position, require general anesthesia. The anesthetic agents result in muscle relaxation and loss of sensation, while prolonged immobilization contributes to cutaneous hypoxia and the accumulation of tissue metabolites. All the factors above lead to skin pressure ulcers (22). The development of pressure increases the risk of infection, causes persistent pain, and prolongs hospital stays, adversely affecting the physical and mental health of patients (23). Therefore, implementing effective preventive nursing procedures is crucial to minimize their occurrence.

This study demonstrated that the gradient warning nursing procedure (GWNP) guided by the Pressure Injury Risk Factor Assessment Scale (PIRFAS) and ultrasonography was associated with improved outcomes across multiple clinical domains. Specifically, the intervention was associated with a markedly lower incidence of pressure ulcers, consistent with previous studies confirming the effectiveness of structured risk assessment in reducing the occurrence of pressure ulcers (24). Furthermore, the joint group showed significant improvements in ulcer severity—including number, area, and grading. These findings not only validate the value of ultrasonography in precise tissue assessment but also highlight its therapeutic potential in actively promoting tissue healing and improving clinical outcomes (25). Beyond clinical outcomes, patient comfort—as reflected in GCQ scores, particularly in physiological and psycho-spiritual dimensions—was significantly enhanced. Existing research reports that predictive nursing care combined with ultrasound technology not only alleviates physical suffering but also reduces psychological distress in patients with extensive severe burns, aligning well with our findings that ultrasound-guided warning care improves both physical and psychological comfort (26).

More importantly, the observed reductions in hospitalization duration and costs underscore the health economic benefits of this integrated approach. Research indicates that ultrasonography can compensate for the limitations of visual assessment, enabling healthcare professionals to accurately identify potential high-risk areas and implement targeted interventions (27). Although direct economic evidence supporting ultrasound in this specific preventive role remains limited, preventing such costly complications through improved assessment aligns well with value-based care principles. Furthermore, while the exact mechanisms warrant further investigation, the observed reductions in hospitalization duration and costs are likely multifactorial. The prevention of pressure ulcers, a known cause of extended hospitalization, undoubtedly contributed to this benefit. However, the standardized, multi-component nature of the intervention—emphasizing proactive risk assessment, structured monitoring, and timely nursing responses—may have streamlined perioperative care more broadly. This could have led to more efficient recovery pathways and reduced overall complication rates, thereby decreasing resource utilization. Although we adjusted for key case-mix variables, the contribution of unmeasured factors, such as nuances in discharge practices, cannot be entirely ruled out.

However, this study has several limitations. First, its single-center design and relatively small sample size may limit the generalizability of the findings. Furthermore, the non-randomized, retrospective nature of the group assignment introduces the potential for selection bias. The intensive ultrasound monitoring in the joint group could also lead to detection bias. Blinding the patients and the operating room staff to the group allocation was not feasible due to the conspicuous nature of the intervention. However, to mitigate detection bias, the primary outcome (pressure ulcer diagnosis) was assessed postoperatively using the same clinical criteria by ward nurses who were blinded to the group assignment, ensuring that this endpoint evaluation was independent of the intraoperative ultrasound monitoring. Additionally, the use of non-concurrent groups raises the possibility of temporal confounding. Although a multivariable regression analysis was performed to adjust for key confounders, residual confounding from unmeasured factors cannot be ruled out. Specifically, the joint group’s intervention is an integrated care bundle, including risk assessment-based gradient early warning, new dressings, standardized posture management, and multi-level quality control. These components form a complete prevention system, making it difficult to quantify the individual contributions of PIRFAS and ultrasound guidance alone or separate them from the bundle’s physical interventions under the current study design. Notably, with a well-defined primary endpoint (pressure ulcer incidence), other outcomes (e.g., ulcer stage, number, area, comfort score, and length of hospital stay) were exploratory; thus, no strict multiplicity correction (e.g., Bonferroni) was applied. Interpretations should focus on effect estimates (e.g., mean difference and odds ratio) and clinical significance rather than solely *p*-values. The absence of a formal mediation analysis prevents us from definitively establishing the extent to which the reduction in pressure ulcers directly mediated the observed shorter hospital stays, as opposed to other aspects of the bundled intervention or external factors. Second, the lack of long-term follow-up limits the assessment of the intervention’s sustained effects.

## 5 Conclusion

In this retrospective cohort study, the gradient early warning care protocol guided by the PIRFAS and ultrasonography was associated with reduced pressure ulcer incidence, less severe tissue damage, improved patients’ comfort and decreased hospitalization duration and costs in patients with prone-positioned orthopedic operations. Future research should prioritize prospective, multicenter, randomized controlled trials to verify the efficacy of this bundled intervention and establish a causal relationship. Furthermore, studies employing factorial designs are warranted to deconstruct the individual contributions of PIRFAS and ultrasonography within the care bundle and to evaluate the long-term impact on patient outcomes.

## Data Availability

The original contributions presented in the study are included in the article/supplementary material, further inquiries can be directed to the corresponding author.
